# The value of continuous nursing in patients after cardiac mechanical valve replacement

**DOI:** 10.1186/s13019-020-01326-5

**Published:** 2020-10-06

**Authors:** Sai-lan Li, Sheng-huo Zhou, Yan-juan Lin

**Affiliations:** Department of Cardiovascular Surgery, Union Hospital, Fujian Medical University, Fuzhou, 350001 Fujian P. R. China

**Keywords:** Continuous nursing, Cardiac valvular disease, Mechanical valve replacement

## Abstract

**Objective:**

The purpose of this study was to explore the value of continuous nursing in patients after cardiac valve replacement.

**Methods:**

The clinical data of 116 patients after cardiac mechanical valve replacement from January 2017 to January 2018 were analysed retrospectively. According to the nursing mode, the patients were divided into two groups: the continuous nursing group (group A, *n* = 56) and the conventional nursing group (group B, *n* = 60).

**Results:**

The continuous nursing group exhibited significantly decreased SAS and SDS scores 1 year after surgery compared to the preoperative SAS and SDS scores(*P* < 0.05). The SAS and SDS scores of the continuous nursing group were significantly better than those of the traditional nursing group 1 year after surgery(*P* < 0.05). There were 4 patients with anticoagulant complications after discharge in the continuous nursing group, and 13 cases of anticoagulant complications in the conventional nursing group. There was a significant difference between the two groups.

**Conclusion:**

Continuous nursing improves patient compliance with treatment and reduces the occurrence of postoperative anticoagulation complications. The patient also receives proper psychological evaluations, which relieve patient anxiety and depression.

## Introduction

In China, cardiac valve disease is the most common acquired heart disease in adults, and cardiac mechanical valve replacement is the main method for clinical treatment of all kinds of cardiac valve disease in China [1]. However, valve replacement requires lifelong anticoagulation therapy postoperation [2]. The behaviour of many cardiac valve replacement patients after discharge is far different from their health behaviour at the hospital. However, the self-management abilities and compliance with treatment decreased with the extension of discharge time, which resulted in anticoagulant deficiency or excess and complications, such as embolism and bleeding, that endangered the life of the patient [3, 4].

Conventional nursing includes systematic education and discharge guidance of disease-related knowledge during hospitalization, and it terminates the health guidance of patients after discharge. Continuous nursing is the extension of high-quality medical services to the family to understand the compliance behaviour, treatment effect and psychological state of the patients after discharge from the hospital and provide the patients with medical and psychological guidance to improve their quality of life [5, 6].

## Method

This study was approved by the ethics committee of our hospital and strictly adhered to the tenets of the Declaration of Helsinki. In addition, all patients or the patient’s relatives signed an informed consent form before the study.

### Patients

The clinical data of 116 patients after cardiac mechanical valve replacement from January 2017 to January 2018 were analysed retrospectively. According to the nursing mode, the patients were divided into two groups: the continuous nursing group (group A, *n* = 56) and the conventional nursing group (group B, *n* = 60). All patients completed the Self-Rating Depression Scale (SDS) and Self-Rating Anxiety Scale (SAS) at admission and 1 year after surgery. All the patients’ general clinical data are shown in Table 1. There were no statistically significant differences between the two groups, which indicates that the two groups were homogeneous and comparable. All of the patients also exhibited independent reading and comprehension abilities.

**Table 1:** Comparison of patients’ general data between the two groups

Item	Group A	Group B	*P* value
Number	56	60	
Age (year)	49.6 ± 8.5	53.2 ± 9.5	0.785
Male/Female	24/32	26/34	0.959
New York classification of cardiac function			
I-II	38	39	0.745
III	18	21	
Cardiac valve			
Mitral valve	25	28	0.918
Aortic valve	19	17	
Tricuspid valve	1	1	
Aortic valve and mitral valve	11	14	
Record of formal schooling			
Junior high school and below	36	42	0.779
senior middle school	16	15	
Universities and above	4	3	
Place of residence: urban / rural	17/39	16/44	0.660

Patients met the inclusion criteria if they underwent cardiac mechanical valve replacement. The following exclusion criteria were used: 1) lack of independent reading ability, 2) lack of communication equipment and inconvenient information communication, 3) postoperative death, or 4) refusal of continuing nursing services and participation in the study.

### Nursing methods

#### Conventional nursing

Routine medical services were provided to patients during hospitalization. The attending physician explained disease-related knowledge and surgical procedures to patients and their families. Nursing staff provided diet knowledge, preoperative preparation, postoperative attention, methods, matters requiring anticoagulant drugs after discharge, regular reviews of prothrombin time (PT) or the international normalized ratio (INR) and other health guidance. For patients with severe emotional disorders, we ask a special psychologist to do psychological intervention.

#### Continuing nursing

On the basis of traditional nursing, continuous nursing issued contact cards at discharge. The contents of the contact card included basic patient information and the telephone number and WeChat of the attending doctor, attending nurse and head of the department. The nurse instructed the patient to join the WeChat group and instructed the patient on the use of the WeChat function correctly and skilfully after surgery. A medical staff member in the group was reminded daily at 19:00 to supervise the patients in the group to take anticoagulant drugs, undergo regular outpatient re-examination, and guide the interactive communication, discussion, consultation and question-answer sessions within the WeChat group. At the same time, psychological counselling and psychological support were carried out for patients in need.

#### Adjustment of warfarin dose

The INR was checked once a week at the 1–2 months after the patient was discharged. The INR was checked once every two weeks at the 3–4 months after the patient was discharged. The INR was checked once every three weeks at the 5–6 months after the patient was discharged. The INR was checked once a month at the 7–12 months after the patient was discharged. The INR was checked once every three month after the patient was discharged 1 year. When the INR is greater than 1.7 and less than 2.3, the dosage of warfarin remains unchanged. When the INR is less than 1.7, the dosage of warfarin increases by 0.625 mg. When the INR is greater than 2.3, the dosage of warfarin decreases by 0.625 mg. If the INR is greater than 3, stop taking warfarin on the same day and ask the doctor to inject 20 mg of VitK1. On the second day, the dosage of warfarin decreases by 0.625 mg.

### Research tools

#### SDS

Zung’s SDS [7] was applied. This scale consists of 20 items, including 10 negative symptoms and 10 positive symptoms. Each question represents a feature of depression. All items together reflect the mood, body discomfort symptoms, spiritual movement, behaviour, and psychological symptoms of patients with depression. The score involves 4 grades. The scores were obtained using the scoring method in ascending order (1 to 4) based on the occurrence frequency of positive symptoms. The rough scores were obtained using the reverse scoring method in descending order (4 to 1) based on the occurrence frequency of negative symptoms. The standard score was obtained by multiplying the scores by 1.25 and rounding off the result. Normally, the upper limit score is 41, and the standard total score is 53. A higher score indicates a more significant depression tendency.

#### SAS

Zung’s SAS [8] was applied. This scale mainly evaluates the subjective feeling of anxiety of the patients, and it is a self-evaluation tool. The SAS is extensively applied in clinics and is characterized by high reliability and validity. Fifteen items are stated with negative words. The scores were obtained using the scoring method in ascending order (1 to 4) based on the occurrence frequency of symptoms. Five items [9, 10] are stated with positive words. The scores were obtained using the reverse scoring method in descending order (4 to 1) based on the occurrence frequency of symptoms. The total score was obtained by adding the scores of all items. The standard score was obtained by multiplying the total score by 1.25 and rounding off the result. The mean value of the standard score is 50. Grade description: < 50 means normal, 50 to 59 means mild anxiety, 60 to 69 means medium anxiety, and ≥ 70 means severe anxiety.

### Statistical analysis

Theorem information is expressed as the means±standard deviation. Independent-samples t-test was adopted for intergroup comparisons. The case number and constituent ratio are used to express the qualitative data. Intergroup comparisons of disordered categorical data were conducted using chi-square tests and Fisher’s exact probability tests. Intergroup comparisons of ordered data were performed using the Wilcoxon rank sum test. *P* < 0.05 indicated that the difference was statistically significant.

## Results

There were no significant differences in SAS and SDS scores between the two groups before surgery. The SAS and SDS scores of the continuous nursing group were significantly decreased 1 year after surgery compared to the preoperative SAS and SDS scores, and the differences were statistically significant. However, the SAS and SDS scores of the conventional nursing group were also lower 1 year after surgery than the scores before surgery, but these differences were not statistically significant. The SAS and SDS scores of the continuous nursing group were significantly better 1 year after surgery than those of the traditional nursing group, and these differences were statistically significant (Table 2).

**Table 2:** Comparison of psychological state before and after nursing intervention

Item	Group A	Group B	P value
The SDS score			
Preoperation	44.8 ± 10.6	45.3 ± 12.7	0.897
1 year after operation	33.8 ± 9.2*	44.1 ± 11.3	0.022
The SAS score			
Preoperation	51.3 ± 13.2	53.2 ± 14.5	0.912
1 year after operation	40.2 ± 10.6*	50.5 ± 12.4	0.018

The “*” indicate that compared with preoperation the *P* < 0.05

All patients were followed up for 1 year. There were 4 patients with anticoagulant complications after discharge in the continuous nursing group, including 3 cases of bleeding and 1 cases of embolism, and 13 cases of anticoagulant complications in the conventional nursing group, including 10 cases of bleeding and 3 cases of embolism. There was a significant difference between the two groups (*P* = 0.027).

## Discussion

Continuous nursing extends treatment and rehabilitation care during hospitalization to the family after discharge to help improve the self-management of patients and ensure that information, treatment and nursing services continue without sudden interruption. Continuous nursing is a new nursing service model that occurred with the development of society and changes in medical services [11, 12]. In China, the current medical services are mainly focused on hospitals, and the relationship between doctors and patients ends when the patients leave the hospital [13].

Mechanical valve replacement is an effective method for the treatment of cardiac valve diseases, and it greatly improves the cardiac function of patients and saves their lives. Most patients who undergo valve replacement in China are middle-aged people. These patients choose a mechanical valve, and they must be treated with lifelong anticoagulant therapy after surgery. Warfarin is the main anticoagulant in China. Therefore, these patients must regularly monitor the INR or PT after surgery and constantly adjust the dose of anticoagulant to prevent bleeding, embolism and other complications of improper anticoagulant treatment. Most of the mechanical valve replacement patients in China are from rural areas, and their academic qualifications are generally low. The lack of disease-related knowledge, poor self-management ability, and the lack of awareness of the importance of anticoagulant therapy result in an inability to take anticoagulant drugs correctly after discharge, which easily causes insufficient or excessive anticoagulant and complications, such as embolism and bleeding [14, 15]. Therefore, we should strengthen the management of patients after valve replacement after discharge, inculcate health education knowledge into patients without interruption and give patients correct guidance and help to avoid the disconnect of the patients from hospitals to the family to solve the problem of insufficient support after discharge. Many studies have shown that continuous nursing can improve patients’ compliance after discharge and reduce complications. Chi S et al. [16] conducted a prospective study to explore the impact of continuous nursing intervention on the rehabilitation of diabetic patients. The results showed that continuous nursing can improve patients’ self-care ability, and reduce the occurrence of complications. Yu YL et al. [17] conducted a randomized controlled study, and the report showed that continuation care can help patients with chronic obstructive pulmonary disease to improve their compliance with oxygen therapy and self-care ability. The study of Li P et al. [18] shown that continuous care can improve the self-efficacy of chronic obstructive pulmonary disease patients and reduce complications. Our study has reached the same conclusion. In the continuous nursing group, we remind patients to review the INR as planned and help them adjust the warfarin dose in time through WeChat. However, many patients in the convention nursing group forget to review the INR regularly, especially for patients with poor compliance. It is also more inconvenient for them to ask doctors to adjust the dosage of warfarin. Therefore, there are fewer complications related to anticoagulation in the continuous nursing group.

Cardiac valve replacement has a relatively high risk of surgical complications compared to other surgeries, and the patient does not possess relevant knowledge of the operation. Therefore, fear and tension are inevitable before and after surgery. Cardiac mechanical valve replacement requires lifelong anticoagulant therapy. For most patients, there will be a heavy psychological burden. Some patients lose confidence, and the negative feelings are obvious. The main causes of anxiety should be evaluated during the diagnosis and treatment process and intervened in a timely manner. If negative emotions are caused by long-term disease remission, the main purpose of the intervention is to improve the main symptoms and comfort patients. If a patient’s anxiety is caused by a lack of understanding of the disease and doubts about the efficacy of the treatment, doctors and nurses should communicate with the patient as soon as possible and explain the process and effect of cardiac valve replacement. If necessary, other similar patients may be invited to share their personal experiences to enhance their confidence. If a patient is worried about their lack of drug and nursing knowledge after discharge, the continuous nursing model with improved WeChat, telephone and other follow-up methods to provide professional service and psychological support to patients after discharge may solve this problem. The services of continuous nursing provide not only professional knowledge and support for the treatment of symptoms but also psychological support to promote the physical and mental recovery of patients [19]. Psychological support is helpful to enhance the patient’s subjective initiative and self-control ability, to meet the safety and comfort needs of patients with special conditions, to alleviate the patient’s undefined negative psychology, to eliminate the patient’s undefined worries, to help patients maintain the best physical and mental state and to improve the patient’s undefined psychological adaptability and quality of life [20]. This study provided a series of continuous nursing services for patients after mechanical heart valve replacement to understand the patients’ needs, difficulties and psychological feelings in a timely manner and to provide effective communication and guidance to solve problems and effectively alleviate the symptoms of depression and anxiety in patients.

There are several limitations in this study. First, this study was a retrospective study with a small sample size, and patients who did not have independent reading ability, communication equipment or convenient information communication were not included in this study, which may cause selection bias. Second, this study was a single-centre study, and more research from multiple centers is mandatory to assess the value of continuous nursing in the future. Third, the follow-up period of this study was brief, and a longer term follow-up period is needed. Fourth, during the follow-up period, the coagulation function re-examination was performed in the local hospital, and the INR measurement method may be different in different hospitals.

## Conclusion

Continuous nursing extends high-quality hospital nursing service and psychological support to the patient’s family, which improved patient compliance with treatment, reduced the occurrence of postoperative anticoagulation complications, and provided the patient with proper psychological evaluation to relieve anxiety and depression.

## Data Availability

The datasets used and analysed during the current study are available from the corresponding author on reasonable request.

## Abbreviations

SDS: Self-Rating Depression Scale
SAS: Self-Rating Anxiety Scale
PT: Prothrombin Time
INR: International Normalized Ratio
